# Feasibility and accuracy evaluation of novel 2D instant navigation system on spinal surgery - a preclinical study

**DOI:** 10.1186/s12891-025-08769-8

**Published:** 2025-05-23

**Authors:** Wen Xia, Zhengyang Wu, Rui Zuo, Jiang Wu, Jing Ling, Linfeng Mo, Zegang Shi, Yue Zhou, Changqing Li, Wenjie Zheng, Chao Zhang

**Affiliations:** 1 Department of Orthopedics, Xinqiao Hospital, Third Military Medical University (Army Medical University), No.183, Xinqiao Main Street, Shapingba District, Chongqing, 400038 China; 2 Chongqing Municipal Health Commission Key Laboratory of Precise Orthopedics, No.183, Xinqiao Main Street, Shapingba District, Chongqing, 400038 China; 3 State Key Laboratory of Trauma and Chemical Poisoning, No.183, Xinqiao Main Street, Shapingba District, Chongqing, 400038 China; 4 R & D Center, Chongqing Boshikang Technology Co., Ltd., No.78, Fenghe Road, Beibei District, Chongqing, 400722 China

**Keywords:** Spine surgery, Intraoperative navigation, Permanent calibration, Two-dimensional fluoroscopic image

## Abstract

**Background:**

Intraoperative navigation has significantly facilitated spinal surgery and enhanced surgical accuracy. Nevertheless, it is often encumbered by the need for expensive equipment, a complex workflow, and frequently exhibits inefficiencies. Leveraging permanent calibration technology, we have developed a novel two-dimensional fluoroscopic image navigation system with the aim of streamlining and expediting the navigation process. In this study, we comprehensively evaluated its feasibility and accuracy.

**Methods:**

The accuracy of the 2D-navigation system was rigorously assessed using a standardized high-precision mold. To validate the feasibility and accuracy of the novel navigation system for spinal surgery, the bare-bones of the pig lumbar spine are employed for evaluation. Subsequently, 2D navigation-assisted pedicle penetrations were meticulously carried out on the spine (L1-L5) of live animals. The navigation accuracy was quantified by comparing the visualized position of the surgical tool in the actual fluoroscopic image with the virtual position pre-planned by the navigation system.

**Results:**

During the experimental process, an excellent correlation between the virtual fluoroscopic images and actual fluoroscopic images was prominently observed. The navigation positioning accuracy, as evaluated by the standardized high-precision mold, was determined to be 0.54±0.16mm (AP view) and 0.57±0.14mm (lateral view). Specifically, in the bare-bones of the pig lumbar spine, the average distance errors between the virtual and actual fluoroscopic images under anteroposterior and lateral views were 0.99±0.48mm and 0.87±0.60mm, respectively. Meanwhile, the average angle errors were 0.41±0.29$^{\circ }$ and 0.37±0.11$^{\circ }$, respectively. In the surgical procedure on normal adult pigs (L1-L5), the average distance errors were 1.14±0.58mm(95% CI [0.50-0.59]) and 1.54±0.79mm(95% CI [0.11-0.12]), respectively. The corresponding average angle errors were 0.61±0.49$^{\circ }$ (95% CI [0.33-0.35]) and 0.40±0.31$^{\circ }$ (95% CI [0.33-0.47]), respectively. Throughout a single navigation registration and the entire surgical procedure, the navigation accuracy across the L1 to L5 segments remained consistently high, with no statistically significant differences detected among the segments (p>0.05).

**Conclusion:**

The two-dimensional fluoroscopic image navigation system based on permanent calibration technology is characterized by a rapid and convenient workflow. It demonstrates high-level navigation accuracy, thereby meeting the stringent requirements for spinal navigation in live surgical procedures.

## Introduction

The deep location and complex structure of spinal surgery, along with its proximity to vital neurological and vascular tissues, have made the safety and precision of spinal surgical manipulation a significant focus in clinical practice [1]. Traditional open spine surgery has clinical problems such as large trauma, slow recovery, and high risk of infection. The application of X-ray imaging (C-arm, G-arm, or O-arm) technologies has enabled surgeons to visualize the patient’s skeletal anatomy, facilitating more accurate placement of pedicle screws [2]. While X-ray imaging has been widely accepted and applied in the field [3], frequent intraoperative imaging will prolong surgical duration and increase radiation exposure for both patients and medical staff, elevating the risk of infection.

Since the introduction of computer navigation technology into spinal surgery, this technology has continuously advanced and innovated. Currently, these technologies are widely used in spinal procedures, providing precise navigation through real-time tracking of surgical instruments in relation to the patient’s anatomical structures. Navigation technology contributes to elevating procedural precision and shortening patient recovery duration, thereby emerging as an indispensable tool for surgeons [4].

At present, in clinical practice, there are two widely used navigation modes in clinical practice for spinal surgery: intraoperative three-dimensional (3D) computed tomography (CT) imaging guidance and intraoperative two-dimensional (2D) fluoroscopy imaging guidance [5, 6]. The former involves acquiring 3D CT images during surgery, combining with spatial registration and positioning methods, to provide surgeons with intuitive and 3D vertebral structure information for auxiliary guidance. The advent of 3D navigation, known for its high accuracy in spinal surgery, has reduced the need for revision surgery [7–11]. Moreover, for patients with severe spinal degeneration, deformities, and trauma resulting in unclear anatomical landmarks, the real-time 3D image guidance is particularly beneficial [12–15], making it the mainstream navigation product. However, it is confronted with clinical challenges, such as low data acquisition efficiency, high radiation dose, high equipment cost, and severe artifact interference. In contrast, the latter mainly relies on intraoperative X-ray equipment to collect anteroposterior (AP) and lateral fluoroscopic images to assist the surgeon in performing the procedure [16–18]. Although it lacks certain 3D spatial structure information, it offers advantages such as convenient intraoperative data acquisition, low fluoroscopy dose, simple operation process, and low economic cost. Obviously, experienced surgeons are more inclined to choose the latter when faced with heavy surgical pressure.

In summary, despite the numerous advantages of 3D navigation in spinal surgery, 2D navigation still has distinct advantages in specific scenarios. One significant advantage is the lower radiation exposure [19]. During spinal surgeries, especially endoscopic and percutaneous pedicle screw placement procedures, minimizing radiation is of crucial importance for both patients and medical staff. Compared with their 3D counterparts, 2D navigation systems generally emit less radiation during the guidance process. Moreover, 2D navigation offers a higher operation speed. It provides real-time guidance in both AP and lateral views. This quick and efficient visual feedback enables surgeons to make prompt decisions, thus reducing the overall surgical time. As a result, 2D navigation offers a more streamlined and effective visual experience during these surgical procedures.

For 2D navigation based on AP and lateral fluoroscopic images, it mainly involves two key processes: calibration and tracking. Tracking can commence only after completing the calibration work. The positioning system captures the spatial position of surgical instruments and displays them in real-time under a unified coordinate system. Currently, the navigation products integrate both calibration and tracking during surgery [16–19], which undoubtedly not only reduces surgical efficiency but also limits the operational space available to the surgeons. For example, Foley et al. [20] first introduced a new technology that combines image-guided surgery with C-arm fluoroscopy, termed “virtual fluoroscopy”, also known as familiar 2D navigation. However, its calibration process is cumbersome and time-consuming. Furthermore, intricate auxiliary devices frequently have to be incorporated to ensure the stability of the navigation system. Inevitably, these elements not only consume operative time but also occupy precious surgical space, thereby leading to a reduction in surgical efficiency and an elevation in surgical risks [21].

Consequently, this research aims to resolve the issues of intricate calibration, time-consuming procedures, and inefficiencies inherent in traditional navigation products. We put forward the proposal of affixing trackers to the X-ray source and the image intensifier. By leveraging a permanent calibration approach, the spatial transformation relationship with the optical tracker can be instantaneously acquired. This facilitates automatic image registration during the capture of fluoroscopic images, thereby enabling instant navigation upon the completion of fluoroscopic shooting. This study assesses the accuracy and feasibility of the instant navigation system grounded in permanent calibration technology.

## Methods

### Navigation system registration process

**Fig. 1:**
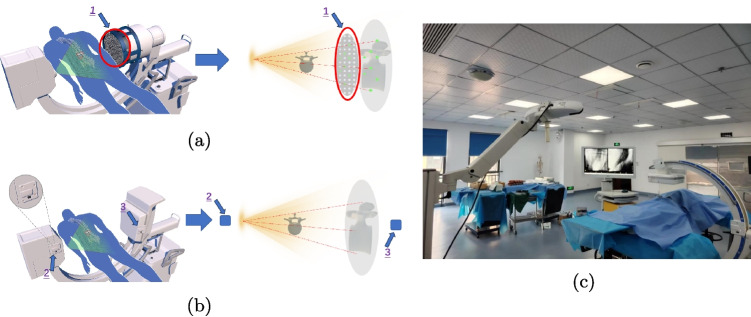
Comparison diagram of different calibration methods for X-ray machine: 1. *phantoms*; 2. *trk-s*; 3. *trk-img*. **a** Schematic diagram of calibration principle of traditional online calibration technology. **b** Schematic diagram of calibration principle of novel permanent calibration technology. **c** Real time fluoroscopic image navigation system under permanent calibration of intraoperative X-ray machine

As shown in Fig. 1a, Regarding the traditional online or offline calibration methods for surgical navigation guided by 2D fluoroscopic images, they have to introduce cumbersome auxiliary devices, such as “phantoms”, “drums”, “rings”, or “dual planes”, to maintain the stability of the navigation system. Meanwhile, in the surgical navigation scenario, the repetitive calibration operations are required for each surgical navigation, which not only causes a constraint on the available surgical operating space, but also results in decreased surgical efficiency and heightened surgical risks.

**Fig. 2:**
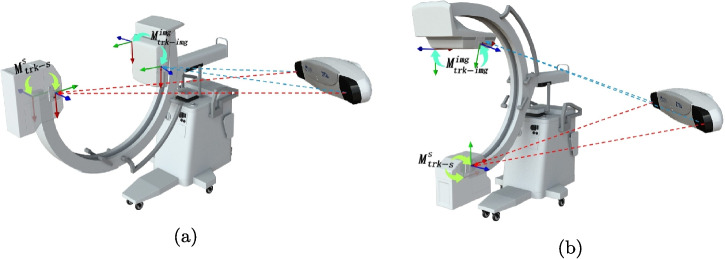
Real-time spatial registration of fluoroscopic images and X-ray sources using permanent calibration method. **a** The lateral fluoroscopic image and X-ray source poses ($M_{cam}^{img}, M_{cam}^{s}$) relative to the optical camera are computed using real-time tracker poses ($M_{cam}^{trk-img}, M_{cam}^{trk-s}$) and pre-calibrated matrices ($M_{trk-img}^{img}, M_{trk-s}^{s}$). **b** When the fluoroscopic angle of X-ray machine changes, the pre-calibrated transformation matrices ($M_{trk-img}^{img}, M_{trk-s}^{s}$) between the fluoroscopic image and the X-ray source tracker remain invariant. Consequently, the lateral fluoroscopic image and X-ray source poses ($M_{cam}^{img}, M_{cam}^{s}$) relative to the optical camera can also be computed in real-time

Unlike the traditional calibration method for surgical navigation, our proposed permanent calibration method requires the use of two self-developed optical trackers, namely X-ray source tracker *trkr-s* and fluoroscopic image tracker (*trkr-img*), which are embedded and rigidly connected to one end of the X-ray source (*S*) and image plate (*Img*) respectively (See Fig. 1b and c). As shown in Fig. 2, the spatial transformation matrices $M_{trkr-s}^{s}$ between *trkr-s* and *S*, as well as $M_{trkr-img}^{img}$ between *trkr-img* and *Img*, are obtained through the permanent calibration algorithm [21]. Specifically, the lateral fluoroscopic image pose $M_{cam}^{img}$ and X-ray source pose $M_{cam}^{s}$ relative to the optical camera are computed by fusing real-time tracker poses $M_{cam}^{trk-img}$ and $M_{cam}^{trk-s}$ with pre-calibrated transformation matrices $M_{trk-img}^{img}$ and $M_{trk-s}^{s}$, as detailed in Fig. 2a. Notably, these pre-calibrated matrices remain invariant despite changes in the X-ray machine’s fluoroscopic angle, enabling continuous real-time computation of $M_{cam}^{img}$ and $M_{cam}^{s}$ under dynamic viewpoints, as demonstrated in Fig. 2b. Therefore, regardless of the posture of the X-ray machine, as long as the navigation system can collect transformation matrices $M_{cam}^{trk-s}$ and $M_{cam}^{trk-img}$, it can obtain real-time spatial information of X-ray source and perspective image relative to optical camera under corresponding perspective angles.

Among them, each tracker is outfitted with four precisely arranged near-infrared optical positioning beads (NIOPB). These beads are distributed in strict compliance with the technical specifications of Northern Digital Inc.’s Polaris Vega ST. This meticulous arrangement ensures that the optical camera can effectively perform target identification and achieve accurate spatial localization. The NIOPB have the following specifications: a power rating of 3 W, an emission angle of 120$^{\circ }$, with the chip fabricated from AlGaAs material. The physical dimensions of the bead are 3.5$\times$3.5 mm^2^, and it operates at a wavelength of 940 NM [21]. Additionally, each tracker must be within the effective spatial range of the optical camera. This effective spatial range is specifically defined as being between 2400 mm and 3000 mm from the origin coordinates of the camera. Among them, the proximal field-of-view area has dimensions of 1313 mm in height and 1566 mm in width, while the distal field-of-view area has dimensions of 1470 mm in height and 1856 mm in width.

On this foundation, by real-time acquiring the position $M_{cam}^{Tool}$ of the surgical tool (*Tool*) whose marking points meet the parameter requirements of Polaris Vega ST with respect to optical camera, the system can integrate the surgical tool, perspective image, as well as the corresponding X-ray source position within the perspective, all within the framework of the camera coordinate system $O_{xyz}^{cam}$ in real-time. Specifically, the surgery was performed using the X-ray instant surgical navigation systems (ZETNa, Chongqing Bosscom Technology Co., Ltd., Chongqing, China) and specialized surgical instruments. Additional, the communication of the aforementioned matrix data adheres to the Socket protocol within the Ethernet framework.

Therefore, optical trackers (*trkr-s* and *trkr-img*) are ingeniously utilized as intermediate media in intraoperative navigation scenes to achieve real-time spatial positioning of the perspective image and its corresponding X-ray source in any posture, once and for all. During the surgical process, only the time for fluoroscopic images transmission and spatial registration is involved. Notably, this combined time will not exceed 10 s, significantly streamlining the workflow and enhancing surgical efficiency [21].

### Navigation accuracy verification on standardized high-precision mold

**Fig. 3:**
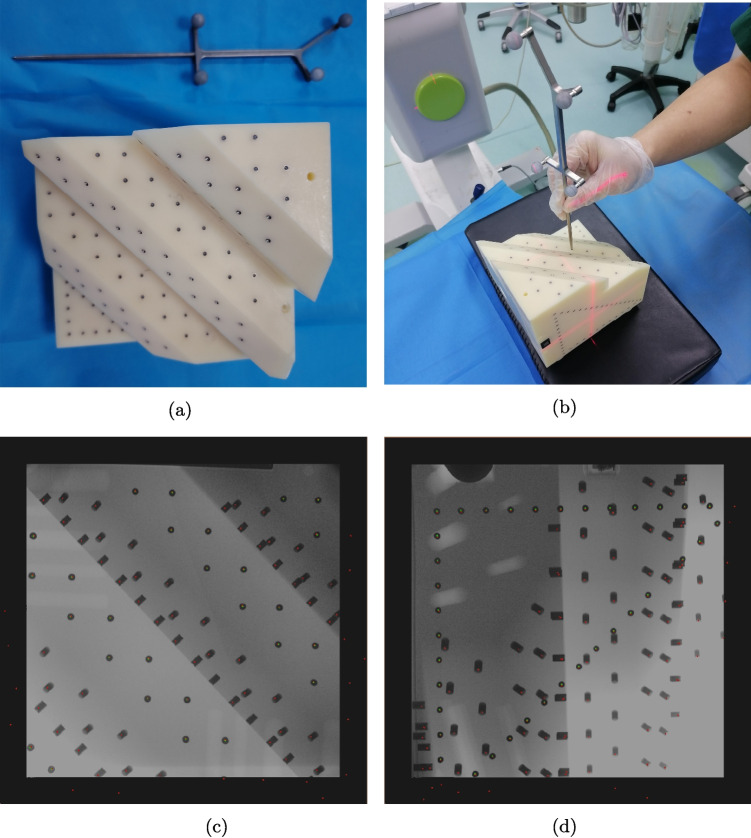
Mold Verification of navigation system accuracy. **a** Physical drawing of high-precision mold. **b** Schematic diagram of the data collection process. **c** Comparison between the actual position (green dots) and the virtual position (red dots) under AP view. **d** Comparison between the actual position (green dots) and the virtual position (red dots) under lateral views

To rigorously validate the accuracy of the navigation system, a custom-fabricated high-precision mold was meticulously designed. The coordinate points of this mold were precisely determined by employing advanced high-precision measuring instruments, ensuring the establishment of a highly accurate reference framework. Four actively-illuminated optical spheres, carefully calibrated and precisely positioned, were embedded within the mold. These spheres served a dual-function: to establish a stable and accurate coordinate system and to facilitate high-precision navigation tracking (Fig. 3a).

Subsequently, an optically-calibrated probe, specifically designed to meet the stringent requirements of high-precision measurement, was utilized. All 78 pre-defined marking points on the mold were individually and precisely probed (Fig. 3b). The optical navigation system was then deployed to record the coordinates of the probe tip within the mold’s coordinate system with sub-millimeter accuracy. Based on the recorded data, the Euclidean distances between the probe tip coordinates and the precise coordinates of the marking points in the mold’s coordinate system were computationally determined. In total, 78 distance values were derived and subsequently subjected to in-depth statistical analysis.

Thereafter, the standardized mold was precisely positioned within the imaging field of the G-arm. AP and lateral fluoroscopic images featuring the marking points were systematically acquired in both the AP and lateral views. From these actual images, accurate and distinct 2D coordinates of the marking points were extracted (38 points in the AP view and 40 points in the lateral view).

Employing the 2D navigation algorithm, the 3D coordinates of the mold were projected onto the fluoroscopic images. The resulting visualized projections are presented in Fig. 3c and d. Subsequently, the distance errors between the coordinates obtained by the cursor and the corresponding projected points were computationally calculated, providing a quantitative measure of the system’s accuracy in the 2D projection scenario.

### Verification of navigation accuracy on bare bone model(L1-L5)

To further comprehensively simulate the accuracy and safety of vertebral navigation in a pre-clinical setting, researchers procured five lumbar vertebrae (L1-L5) from adult pigs with a body weight ranging from 120 to 130 kg. All surrounding muscle tissues and ligaments were meticulously excised, and partial spinous and transverse processes were carefully removed. Subsequently, the vertebrae were firmly fixed on the surgical table in a position with the spinous processes oriented upward. This fixation method was designed to ensure absolute stability of the bones, preventing any inadvertent movement during the experimental procedures.

The reference frame was strategically and precisely positioned in relation to the fixed vertebrae. A high-resolution scan was then executed using a G-arm imaging device to acquire comprehensive imaging data for both the reference frame and the surgical area. The AP and lateral fluoroscopic images were promptly and accurately transmitted to the navigation host system for intricate registration and alignment processes. These processes were crucial for establishing a precise spatial relationship between the real-world anatomical structures and the virtual representation within the navigation system.

**Fig. 4:**
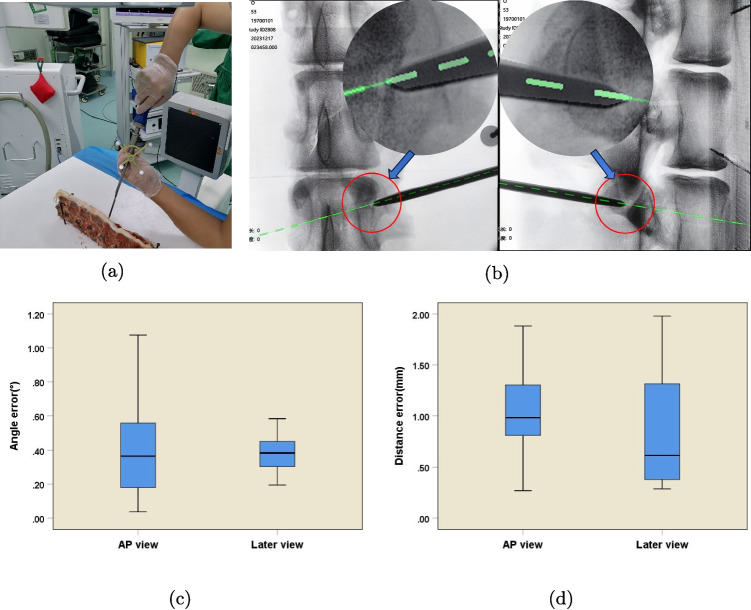
Verification of navigation accuracy on bare-bone of pig spine. **a** Pedicle punctures guided by navigation on pig bone. **b** Superimposition of the virtual and actual probes. The correlation is excellent. **c** The angle error distributions with a median angle error of 0.36$^{\circ }$ (IQR: 0.14–0.56$^{\circ }$) in the AP view and 0.38$^{\circ }$ (IQR: 0.27–0.45$^{\circ }$) in the lateral view. **d** The distance error distributions with a median distance error of 0.98 mm (IQR: 0.64–1.32 mm) in the AP view and 0.61 mm (IQR: 0.35–1.35 mm) in the lateral view. IQR: interquartile range

Following the successful registration, 15 guided punctures were systematically carried out from L1 to L5. This sequential puncturing approach allowed for a detailed assessment of the navigation system’s performance across multiple vertebral levels. After the puncturing procedures, the actual fluoroscopic position of the navigation tool was meticulously compared and measured against the virtual position displayed by the navigation system (Fig. 4a and b). This comparison provided a direct and quantitative evaluation of the navigation system’s accuracy in a clinically relevant scenario, enabling researchers to identify any potential deviations and assess the system’s safety and reliability.

### Verification of navigation accuracy on the live-pigs (L1-L5)

The experimental pig was procured from Chengdu Dashuo Experimental Animal Co., Ltd., accompanied by a valid experimental animal certificate. The present study was duly approved by the Animal Ethics Committee of the Army Medical University. The spinal model of miniature pigs has a close resemblance to the anatomical structure of the human spine, as substantiated by previous research [22–26]. In this experiment, three healthy six-month-old male Guangxi Bama minipigs ($n=3$) with a body weight of approximately 18 kg was selected.

**Fig. 5:**
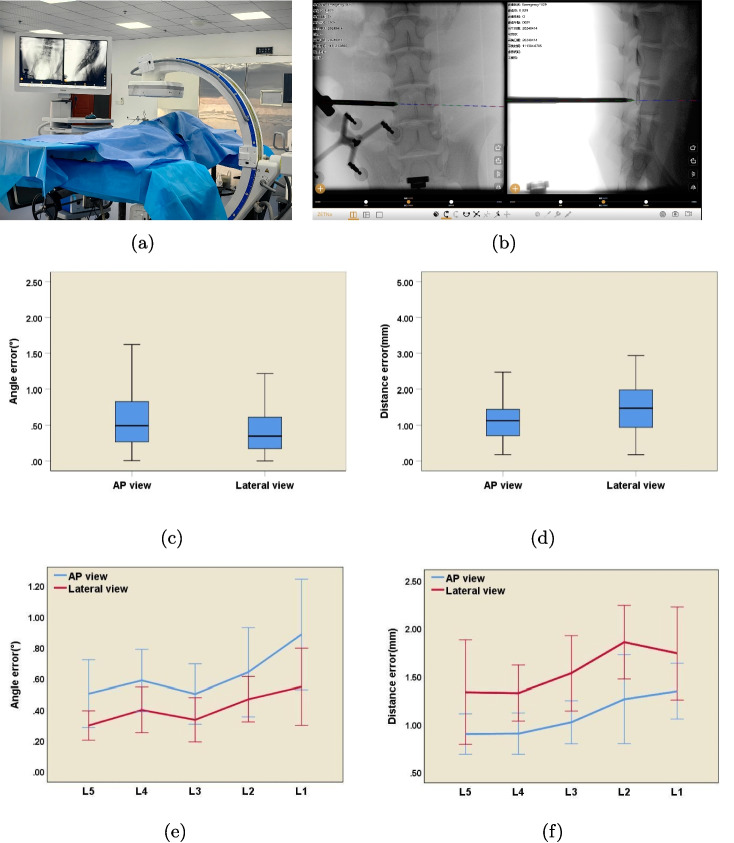
In vivo verification of navigation accuracy in pig lumbar spine (L1-L5). **a** Intraoperative fluoroscopic imaging (AP and lateral views) was acquired for navigation accuracy verification. **b** The virtual pedicle probe superimposed on the L4 vertebra. **c** The angle error distributions with a median angle error of 0.49$^{\circ }$ (IQR: 0.26–0.83$^{\circ }$) in the AP view and 0.34$^{\circ }$ (IQR: 0.17–0.61$^{\circ }$) in the lateral view. **d** The distance error distributions with a median distance error of 1.12 mm (IQR: 0.70–1.44 mm) in the AP view and 1.47 mm (IQR: 0.94–1.98 mm) in the lateral view. **e** Trend of the L1-L5 intervertebral angle error, with AP view 0.61±0.49$^{\circ }$ (95% CI [0.33–0.35]) and lateral view 0.40±0.31$^{\circ }$ (95% CI [0.33–0.47]). The error increases progressively from L5 to L1, but there is no significant difference in accuracy between vertebrae (p>0.05). **f** The trend of the L1-L5 intervertebral distance error, with AP view (1.14±0.58 mm, 95% CI [0.50–0.59]) and lateral view (1.54±0.79 mm, 95% CI [0.11–0.12]). The error increases progressively from L5 to L1, but there is no significant difference in accuracy between vertebrae(p>0.05). IQR: interquartile range; CI, confidence interval; Error bars represent standard deviation ($n=18$)

For anesthesia, 10 milliliters of pentobarbital sodium was intravenously administered to the pig. Once the anesthetic took full effect, the pig was carefully positioned in the prone position on a custom-designed surgical table and firmly secured to prevent any inadvertent movement during the experimental procedures. Standard aseptic disinfection protocols were then meticulously carried out. An optical camera was precisely mounted at the head of the surgical table to capture high-resolution images of the surgical area. Subsequently, a C-arm was accurately positioned beneath the customized surgical table (Fig. 5a).

A pelvic reference frame was rigidly fixed to the corresponding cortical bone. High-quality imaging data for both the reference frame and the surgical area were then systematically acquired using the C-arm. Once the data collection was successfully completed, the C-arm scan data were promptly and accurately transferred to the navigation workstation for complex registration and alignment processes. These processes were essential for establishing a precise spatial correspondence between the real-world anatomical structures and the virtual model within the navigation system.

Continuous percutaneous pedicle punctures were sequentially performed on the pig from L1 to L5. The surgical instrument was precisely inserted into the optimal entry point of the pedicle, the posterior edge of the vertebral body, and further into the bone structure of the vertebra. The position of the puncture instrument was continuously monitored and recorded using C-arm scans (Fig. 5b).

Throughout the study, strict adherence to the ARRIVE guidelines was maintained. Prior to the commencement of any experimental procedures, a detailed experimental protocol was meticulously designed to ensure both the scientific validity and ethical integrity of the study. In terms of animal housing, the Guangxi Bama miniature pigs were provided with a clean, spacious, and well-ventilated environment, meeting all the requirements for the well-being of the animal. During the entire experiment, every effort was made to minimize pain and distress experienced by the animal. All surgical procedures were performed by highly trained and skilled personnel with utmost care. Upon completion of the experimental procedures, euthanasia was conducted by trained personnel. Animals received a single intravenous administration of sodium pentobarbital at a dosage of 100 mg/kg, which rapidly induced deep anesthesia and subsequently led to cardiac arrest.

## Results

In the context of the rapid development of medical technology today, the accuracy of surgical navigation systems plays a pivotal and decisive role in enhancing surgical success rates and reducing surgical risks, and has become the focus of attention in the field of clinical surgery. This study focuses on a new surgical navigation system and conducts in-depth investigations into its accuracy performance under different complex scenarios through a series of scientific, rigorous, and comprehensive experimental methods. The aim is to provide solid data support and reliable theoretical basis for the future clinical application and further optimization of this system. The following will elaborate in detail on the important achievements of this study from three key dimensions: standard-mold validation, bare-bone validation, and live-pigs validation.

Standard-mold validation: In the initial stage of the research, a standardized high-precision mold that has undergone strict calibration was adopted to verify the basic performance of the navigation system. This part of the study focused on obtaining the tracking accuracy of the system. For the AP and lateral views, professional measurement tools and data analysis methods were used to conduct meticulous error quantification analysis. In a navigation system environment with permanent calibration, the average distance error in the AP view is 0.54±0.16 mm, and the average distance error in the lateral view is 0.57±0.14 mm. The experimental results of this stage initially revealed the accuracy level of the navigation system under ideal mold conditions, laid a solid foundation for subsequent validation experiments in more complex and realistic surgical environments, and defined the accuracy benchmark of the system under basic conditions, which is of great reference value.

Bare-bone validation: To further simulate the actual operation scenarios related to bones during surgery, this study carried out the bare-bone validation experiment. The bare bones of the pig spine were selected as the research object. In the L1-L5 segment, 15 puncture operations were carefully carried out in accordance with strict experimental operation procedures. During each puncture, three key anatomical position points, namely the entry point of the articular process, the posterior margin of the vertebral body, and the internal bony structure of the vertebral body, were strictly selected as measurement references according to anatomical standards. Using the classical Euclidean geometry theorem, the absolute value of the coordinate difference between the virtual position and the actual position was calculated accurately, and professional data analysis software was used for processing to determine the navigation accuracy. The experimental data showed that under AP view, the average trajectory angle error between the virtual and actual probes was 0.41±0.29$^{\circ }$, and the average distance error was 0.99±0.48 mm; under lateral view, the average trajectory angle error was 0.37±0.11$^{\circ }$, and the average distance error was 0.87±0.60 mm (Fig. 4c and d). The results of the bare-bone validation experiment further verified the accuracy performance of the navigation system in an environment similar to the real bone structure, provided crucial experimental evidence for evaluating the system’s ability to handle bone-related operations in actual surgeries, and helped to deeply understand the performance characteristics of the system in such scenarios.

**Table 1:** Accuracy comparison across vertebral levels

Error	L5	L4	L3	L2	L1	p-value	Mean Difference [95% CI]
Distance error(mm)							
AP view	0.89±0.38	1.03±0.58	1.09±0.51	1.29±0.75	1.42±0.52	0.057	1.14 [0.50–0.59]
Lateral view	1.33±1.02	1.32±0.54	1.53±0.73	1.85±0.66	1.73±0.87	0.117	1.54 [0.11–0.12]
Angle error ($^{\circ }$)							
AP view	0.50±0.43	0.58±0.39	0.49±0.37	0.63±0.53	0.88±0.67	0.346	0.61 [0.33–0.35]
Lateral view	0.29±0.19	0.41±0.28	0.33±0.27	0.45±0.27	0.52±0.45	0.167	0.40 [0.33–0.47]

Values are presented as mean ± standard deviation; *CI* confidence interval

Live-pigs Validation: In order to restore the real surgical environment to the greatest extent and make the experimental results more clinically relevant, this study carried out the live-pigs validation experiment. In the L1-L5 segment of live pigs, 30 puncture operations were performed on each pig in an orderly manner following the ethical norms of animal experiments. Similarly, based on the three key position points of the entry point of the articular process, the posterior margin of the vertebral body, and the internal bony structure of the vertebral body, professional surgical instruments and navigation equipment were used to carry out puncture and accuracy calculation. The experimental results showed that during AP view, the average trajectory angle difference between the virtual and actual probes was 0.61±0.49$^{\circ }$ (95% CI [0.33–0.35]), and the average distance error was 1.14±0.58 mm(95% CI [0.50–0.59]); during lateral view, the average trajectory angle difference was 0.40±0.31$^{\circ }$ (95% CI [0.33–0.47]), and the average distance error was 1.54±0.79 mm(95% CI [0.11–0.12]) (Fig. 5c and d). In addition, a comprehensive evaluation of the accuracy of the navigation system for different vertebral bodies was carried out using statistical methods. No statistically significant difference was observed in accuracy between vertebral bodies (P>0.05). The results clearly showed that the accuracy of the system remained basically stable (Fig. 5e, f and Table 1). The live-pigs validation experiment comprehensively and realistically demonstrated the accuracy of the navigation system in live organisms, provided highly persuasive and valuable experimental data for its subsequent clinical application, and strongly promoted the transformation process of the navigation system from laboratory research to clinical application.

## Discussion

In the ever-evolving landscape of spinal surgery, the relentless pursuit of minimally invasive, digital, and intelligent solutions has become an unwavering objective, driven by the twin forces of technological advancements and the escalating demands of patients [27]. Historically, the calibration of X-ray machine geometric parameters during surgery relied on specialized calibration devices, which not only complicated the surgical field but also significantly extended the surgical duration. A recent study [28] even reported a substantial increase in surgical time compared to non-navigation surgeries. This has created an urgent imperative for the development of real-time, efficient navigation technologies.

In response to these challenges, we have engineered a novel 2D navigation system underpinned by permanent calibration technology. This innovative approach, in tandem with our self-developed optical tracker, enables rapid and pinpoint-accurate spatial positioning during surgery, regardless of the X-ray machine’s spatial orientation relative to the fluoroscopic image and X-ray source [21].

Traditional X-ray equipment calibration methods, namely online and offline calibration [16–19], have inherent limitations. Online calibration, which involves real-time optimization of the X-ray source and image plate’s spatial positioning information corresponding to the intraoperative fluoroscopic image [19, 29, 30], not only elongates the navigation process but also distorts the structural information within the fluoroscopic images. Offline calibration, conducted prior to surgical navigation using specific calibration devices to obtain fluoroscopic images (excluding patient-specific anatomical details) [31], requires dual-perspective scans and a calibration operation for each intraoperative navigation instance, thereby increasing surgical time in clinical settings. In stark contrast, our proposed permanent calibration technology requires only a single calibration, fulfilling the navigation needs for multiple subsequent surgeries [21], which represents a significant leap forward in simplifying the surgical workflow and conserving valuable surgical time and space.

Our navigation system can achieve instant image registration during fluoroscopy, with the entire process completed within a matter of seconds. This is a remarkable improvement over the past, where registration times typically spanned from a few minutes to as long as 10 minutes [32–34]. The operation of this system is as straight forward as conventional fluoroscopy, with one-click data transfer, necessitating minimal training for operators and reducing the need for specialized personnel assistance. Compared with existing navigation systems in the same category, our system demonstrates remarkable efficiency in image registration, with the process completed within seconds, and features a highly straightforward operation similar to conventional fluoroscopy. These characteristics endow it with distinct advantages in terms of speed and convenience, making it a highly competitive option in the field.

Initial assessments using a standardized mold demonstrated that the average distance error in the AP view is 0.54±0.16 mm, and the average distance error in the lateral view is 0.57±0.14 mm. Expanding the evaluation to include the bare-bone of the pig spine (L1-L5) and in-vivo animals spine (L1-L5), the system continued to exhibit remarkable precision. In the bare-bone model experiment, the average distance errors between virtual and actual fluoroscopic images under the anteroposterior and lateral views were 0.99±0.48 mm and 0.87±0.60 mm, respectively, while the average angular errors were 0.41±0.29$^{\circ }$ and 0.37±0.11$^{\circ }$, respectively. In the normal adult pigs (L1-L5) experiment,the average distance errors were 1.14±0.58 mm(95% CI [0.50–0.59]) and 1.54±0.79 mm(95% CI [0.11–0.12]), and the average trajectory angle errors were 0.61±0.49$^{\circ }$ (95% CI [0.33–0.35]) and 0.40±0.31$^{\circ }$ (95% CI [0.33–0.47]). Considering the unique anatomical structure of the pedicle, and based on the typical screw diameters used in different cervical, thoracolumbar segments, the maximum allowable accuracy deviation range for pedicle screw implantation spans from 0 mm and 0$^{\circ }$ for the T5 vertebra to 3.8 mm and 12.7$^{\circ }$ for the L5 vertebra [35–38]. Evidently, our system’s accuracy comfortably meets these stringent requirements, underscoring its high-level accuracy and safety.

Compared to 3D navigation systems that often rely on intricate imaging techniques and consequently result in elevated radiation doses [39, 40], our 2D system effectively mitigates radiation exposure by integrating 2D fluoroscopic images with advanced optical tracking technology. This not only safeguards patient health but also has far-reaching implications for reducing the overall radiation burden in medical settings. In terms of cost-effectiveness, when pitted against robotic-assisted surgical navigation systems, our novel 2D surgical navigation system enjoys a distinct advantage. Robotic-assisted systems are not only burdened with high purchase and maintenance costs but also demand specialized training for surgeons and staff. In contrast, our system, which capitalizes on existing 2D fluoroscopic images, is more cost-effective and more readily adoptable by a wide spectrum of medical institutions.

This study has preliminarily confirmed the accuracy of the novel 2D navigation system based on the new algorithm and simplified the calibration process. However, during protracted surgical procedures, the accuracy and stability of the navigation system are susceptible to a multitude of factors. Accidental contact with the reference frame during operation, intraoperative image drift induced by patient position changes or respiration, and contamination of reflective balls by blood and smoke are merely a few examples of potential disruptors that could undermine the system’s performance. Meanwhile, the successful clinical application of this system in human surgeries highly depends on the operator’s proficiency in traditional intraoperative fluoroscopy. Inexperienced operators may have difficulties in fully exerting the system’s capabilities, potentially compromising surgical outcomes. Additionally, since the system is only calibrated for the fluoroscopy system and lacks comprehensive 3D image information, its use remains confined to conventional fluoroscopy-guided procedures. While the system demonstrates competitive accuracy in tool positioning, it falls short in complex spinal surgeries requiring a holistic 3D anatomical view, such as deformity corrections. Furthermore, anatomical differences between pig and human spines, including smaller endplates, larger pedicles, taller and narrower vertebral bodies, narrower spinal canals, and shorter spinous processes in pigs [41], may lead to underestimation or amplification of navigation errors when extrapolating pig experimental results to human clinical applications. Respiratory motion patterns under general anesthesia also differ between species, further complicating extrapolation. Although our 2D navigation system enhances surgical workflow by providing real-time multi-plane reconstruction by combining virtual fluoroscopy with c-arm image, the lack of axial anatomical imaging limits its utility in upper cervical pathologies, scoliosis, and spinal tumor cases. Thus, mastery of traditional fluoroscopy remains integral for surgeons, particularly for tasks like puncture point localization and pedicle wall breach assessment via AP and lateral views. Nevertheless, the system’s high compatibility with conventional fluoroscopy allows surgeons to rapidly adapt to its use with targeted training. Given these limitations, further clinical trials are essential to rigorously evaluate the system’s efficacy in humans and benchmark it against existing technologies.

Compared to traditional 2D navigation systems (probe tip error: 0.97±0.40 mm; trajectory angle error: 2.7±0.6$^{\circ }$) [3], our system demonstrates superior accuracy, enabling more precise surgical interventions while reducing instrument misplacement risks. Importantly, it minimizes radiation exposure compared to conventional 3D navigation systems. Literature reviews show no statistically significant difference in the overall operative time between the three modalities (218±14.94 min for O-Arm, 210.83±11.38 min for 3D-C-Arm or 193±17.24 min for 2D-fluoroscopy cases [12, 42]). A potential reason for the increased operative duration was the time needed to set up the navigation equipment in the operating room and maintain a sterile technique. Although our 2D navigation system remains in preclinical evaluation, its design significantly reduces fluoroscopy times, provides real-time guidance, and simplifies registration compared to traditional fluoroscopy, which may effectively save the operation time. In the cost-benefit analysis, our system offers a clinically practical solution for minimally invasive spinal surgery, outperforming robotic-assisted systems in affordability while maintaining compatibility with standard C-arm/G-arm fluoroscopy. Requiring only a single preoperative scan for procedures like decompression and pedicle screw placement, it streamlines workflow and eliminates repeated imaging through dynamic tool registration. These advantages in accuracy, radiation efficiency, and cost-effectiveness make our 2D navigation system a promising alternative for widespread spinal surgery, particularly in lumbar degenerative diseases where anterolateral image-guided techniques are routinely employed.

In summary, this study has laid the foundation for validating the accuracy of the novel 2D navigation system based on a new algorithm and has streamlined the calibration process. Although several limitations exist, future research into 2D-3D cross-modal registration technology, along with the conduct of clinical trials, holds the promise of further enhancing the system’s performance. This, in turn, could propel spinal surgery towards a new era of enhanced minimally invasive techniques, intelligence, and patient-centered care.

## Conclusion

In the pre-clinical study, the novel 2D instant navigation system has successfully achieved its key objectives. Developed to address the issues of complex calibration and high radiation in traditional spinal surgery navigation, this system has demonstrated remarkable accuracy, low radiation exposure, and stability. Its average distance errors reached 0.54±0.16 mm (AP view) and 0.57±0.14 mm (lateral view) on the standard-mold, and maintained a high level of precision in subsequent animal experiments, meeting the strict requirements of spinal surgeries. This significantly reduces the risk of surgical instrument misplacement and improves the surgical success rate. By integrating 2D fluoroscopic images with advanced optical tracking technology, the system effectively reduces radiation exposure. This not only protects patient health but also alleviates the radiation burden in the medical environment, making a significant contribution to the field of minimally invasive spinal surgery. Despite potential challenges after a large number of surgical procedures, the system’s stability reflects its potential for practical applications. It offers a familiar operation mode for surgeons accustomed to fluoroscopy, enhancing surgical efficiency. This study validates the accuracy of the novel 2D navigation system based on a new algorithm and simplifies the calibration process.

## Data Availability

Data is provided within the manuscript.The datasets used and analyzed during the current study are available from the corresponding author on reasonable request.
